# A novel flow-based geometrical upscaling method for representing fault zones with two-phase fault rock properties into a dynamic reservoir model

**DOI:** 10.1038/s41598-021-99024-2

**Published:** 2021-09-30

**Authors:** Md Saiful Islam, Tom Manzocchi

**Affiliations:** 1 Department of Mechanical and Mechatronics Engineering, College of Engineering, Dhofar University, Salalah, Oman; 2 Fault Analysis Group and Irish Centre for Research in Applied Geoscience (ICRAG), UCD School of Earth Sciences, University College Dublin, Dublin, Ireland

**Keywords:** Geodynamics, Geophysics, Solid Earth sciences, Energy science and technology

## Abstract

Faults are generally represented in conventional upscaled models as 2D planar surfaces with transmissibility multipliers used to represent single-phase fault properties. However, faults are structurally complex 3D zones in which both single-phase and two-phase fault rock properties can be significant. Ignoring this structural and petrophysical complexity within faults may impart considerable inaccuracy on the predictive performance of upscaled models. This study has developed a two-phase flow-based geometrical upscaling method capable of representing simultaneously the complex geometry and saturation-dependent two-phase flow properties of realistic fault zones. In this approach, high-resolution sector models are built of small portions of the fault zones and assigned appropriate single-phase and two-phase fault rock properties. Steady state two-phase flow simulations at different fractional flows of oil and water are used to determine the saturation dependent upscaled pseudo relative permeability functions which are incorporated into upscaled models. The method is applied to an example model containing two 3D fault zone components and tested by comparing the flow results of upscaled model with those of a high-resolution truth model. Results show that two-phase flow-based geometrical upscaling is a promising method for representing the effects of two-phase fault rock properties and complex 3D fault zone structure simultaneously.

## Introduction

**Figure 1:**
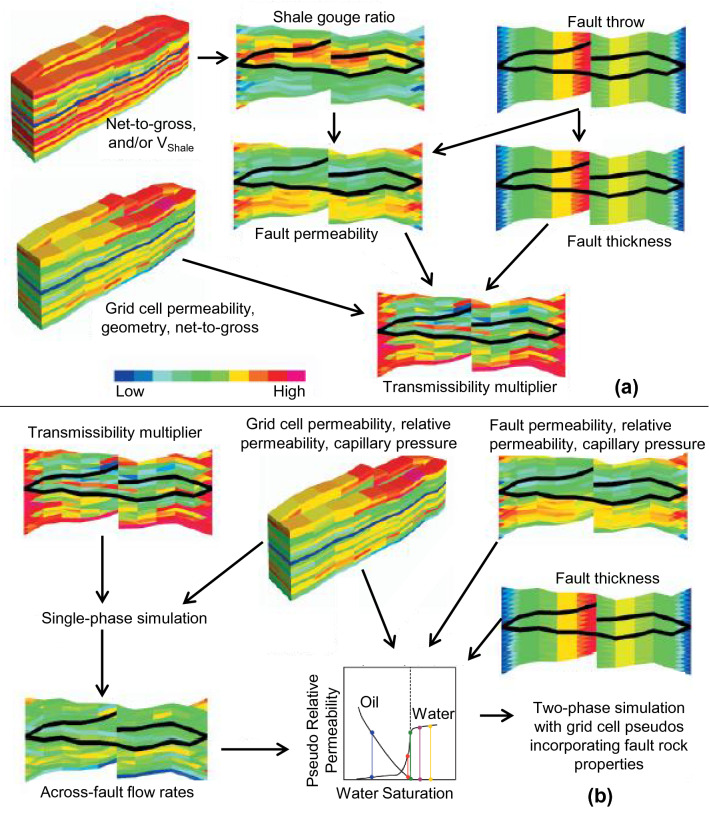
Workflow for incorporating (**a**) single-phase and (**b**) two-phase fault rock properties into flow simulation models. After Manzocchi et al.^1,7,11^.

This study is concerned with developing methods for modeling the effect of fault zones, and their associated fault rocks, on fluid flow in hydrocarbon reservoirs. Both single-phase and two-phase fault rock properties sometimes need to be considered in flow modeling. The single-phase fault rock properties of interest in production flow simulation models of conventional clastic reservoirs are the absolute permeability and thickness of the fault rock. A geologically meaningful method to include these properties as transmissibility multipliers was developed by Manzocchi et al.^1^ and is now applied routinely in flow simulation modeling^2–4^ (Fig. 1a). A key limitation of this method is that it does not take two-phase fault rock properties into account^5–8^. Two-phase fault rock properties (i.e., relative permeability and capillary pressure functions) have been measured in the laboratory^5,8,9^, and several studies have suggested that it may sometimes be necessary to include them in flow simulation modeling to obtain more accurate results^5,7,10–14^.

Two-phase fault rock properties are difficult to include in a flow simulation model because they depend on the water saturation of the fault rock which changes over the course of the simulation run. Since faults are usually included in flow models as zero-volume interfaces between grid-cells, there is no explicit definition of water saturation to which the two-phase fault rock properties can be indexed and this is the source of the difficulty in including them^7^. Attempts have been made to circumvent this restriction. For example, Al-Busafi et al.^5^ presented a workflow to represent two-phase fault rock properties using local grid refinements and have shown that two-phase fault rock properties are more influential than the single-phase ones for a compartmentalized gas reservoir produced by gas expansion. Zijlstra et al.^14^ introduced a simple approach called "capillary entry height" method to incorporate two-phase effects by modifying single-phase multipliers. This method was developed for gas reservoirs in the Southern North Sea produced by gas expansion and is inappropriate for oil reservoirs produced by waterflooding. The present study applies the method developed by Manzocchi et al.^7,11^, in which the effects of two-phase fault rock properties are included in directional, irreversible upscaled relative permeability functions attached to the grid-blocks adjacent to faults. This method is summarized in Fig. 1b.

In addition to the usual practice of omitting two-phase fault rock properties, another simplifying aspect of fault inclusion in conventional flow simulation models is the representation of faults as single planar surfaces accommodating all the fault offset. In reality, fault zones are complicated three-dimensional volumes containing numerous subordinate fault segments each accommodating a portion of the total fault offset^15,16^. The intervening fault lenses are generally too small to be imaged on seismic but can provide across-fault and along-fault flow paths that are not present if the fault is simplified to a single surface. In the case of laterally compartmentalized or vertically heterogeneous reservoirs with low k_V_/k_H_ ratios, these may significantly affect reservoir production^11,17^. Two types of approach have been proposed to represent three-dimensional fault zone structure in full-field flow simulation modeling: representation of the fault zone geometries using local grid refinements^18^ or representation by upscaling^11^. This study considers a type of upscaling called “geometrical upscaling”, which is defined as the process of capturing the transmissibilities of all the tortuous flow paths within, across and along the components of the fault zone, and representing them in the simulation model as neighbor and non-neighbor connections^10,11,19,20^.

**Figure 2:**
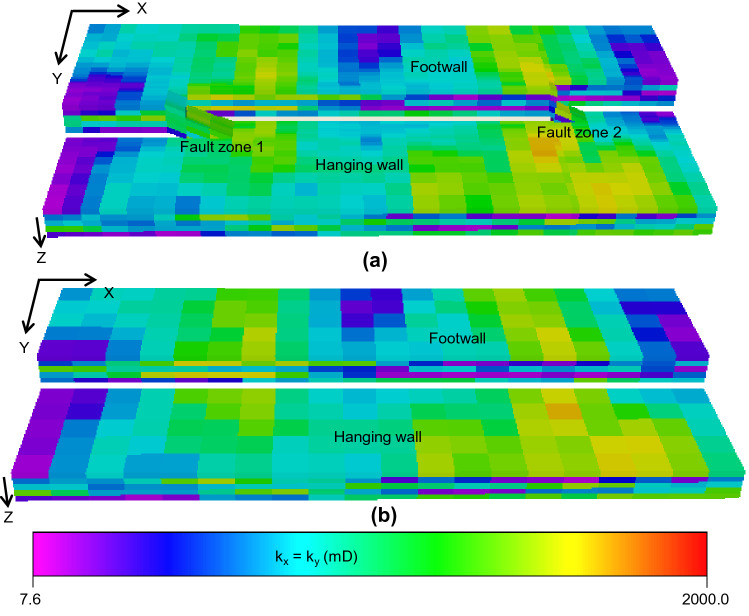
Reservoir models (color bar represents horizontal permeability profile). (**a**) The truth model. (**b**) The upscaled model.

The effects of two-phase fault rock properties and 3D fault zone structure are widely recognized to be potentially significant in reservoir management studies, but, as discussed above, representation of the features using two-phase upscaling and geometrical upscaling respectively, is technically challenging. Both these types of up-scaling have previously been applied separately in full-field simulation modeling^11^, but combining them to upscale two-phase fault rock properties and fault zone structure simultaneously via two-phase geometrical upscaling has not previously been attempted. This paper addresses the technical challenges associated with doing this using a simple model of a single fault (Fig. 2). First, a truth model is constructed (Fig. 2a). This model contains explicitly in the grid structure the two regions of fault zone complexity where communication from one side of the model to the other is created. Individual across-fault connections in the truth model contain low permeability single-phase fault rock properties represented as transmissibility multipliers, and two-phase fault rock properties represented as directional irreversible relative permeability functions calculated using the two-phase fault rock upscaling method discussed above (Fig. 1b). Construction of the high-resolution truth model is described in the next section of the paper.

The objective of the study is to upscale the fault zone in the truth model into a simpler representation as a single continuous surface with the same aggregated throw but none of the geometrical complexities: this is referred to as the upscaled model (Fig. 2b). The two-phase upscaling approach derived in this paper is a development upon the single-phase flow-based geometrical upscaling algorithm developed by Islam and Manzocchi^10^. The new procedure calculates two-phase pseudo-relative permeability curves for each across-fault or up-fault flow path present in the truth model but expressed for the simplified geometrical framework of the upscaled model. Often, the motivation for upscaling is to reduce the number of cells in a model and hence to reduce the CPU time required to simulate it. In this case the situation is different, since it is currently unfeasible to represent fault zone structure and two-phase fault rock properties explicitly in a full-field model. Therefore, the motivation of this study is not to reduce CPU time, but to allow for the inclusion of potentially significant features flow model of faulted clastic reservoirs. Development of the new two-phase flow-based geometrical upscaling method is the main innovation of the paper and is described comprehensively in the method section at the end of the paper. The flow responses of the truth model are compared to those of the upscaled model under a water-flood to check the accuracy of the new upscaling method.

## The high-resolution truth model

This study focuses on a synthetic model of a 500 m long portion of a fault with a throw of 40 m offsetting a 25 m thick reservoir sequence (Fig. 2, Table 1). The high-resolution truth model (Fig. 2a) contains two fault zone components which provide across-fault flow paths between the hanging wall (downthrown side) and footwall (upthrown side) that are absent in the low-resolution upscaled model in which the fault is represented as a continuous surface (Fig. 2b). As well as geometrical complications provided by these fault zones, the fault rocks in the truth model are assigned both single-phase and two-phase fault rock properties using the procedures outlined in Fig. 1. The upscaled model (Fig. 2b) represents the fault as it would appear in a conventional full-field simulation model (i.e., as a single surface) and the overall objective of the study is to upscale the flow paths present in the truth model for representation in this low-resolution upscaled model. A description of the upscaling is provided later; this section describes the construction of the truth model.

**Table 1:** The geometrical structure, volume of interest (i.e., model dimension), and grid-cell dimension of the models.

Truth model (Fig. 2a)	Upscaled model (Fig. 2b)
Contains 3D sub-seismic fault zone components explicitly with their associated single-phase and two-phase fault rock properties	Contains 3D sub-seismic fault zone components and associated fault rock properties upscaled using two-phase flow-based geometrical upscaling
Non-uniform grid-cells	Uniform grid-cells
Model dimension: 500 × 250 × 25 m	Model dimension: 500 × 250 × 25 m
Grid-cell dimension: 30 × 38 × 5	Grid-cell dimension: 20 × 10 × 5

**Figure 3:**
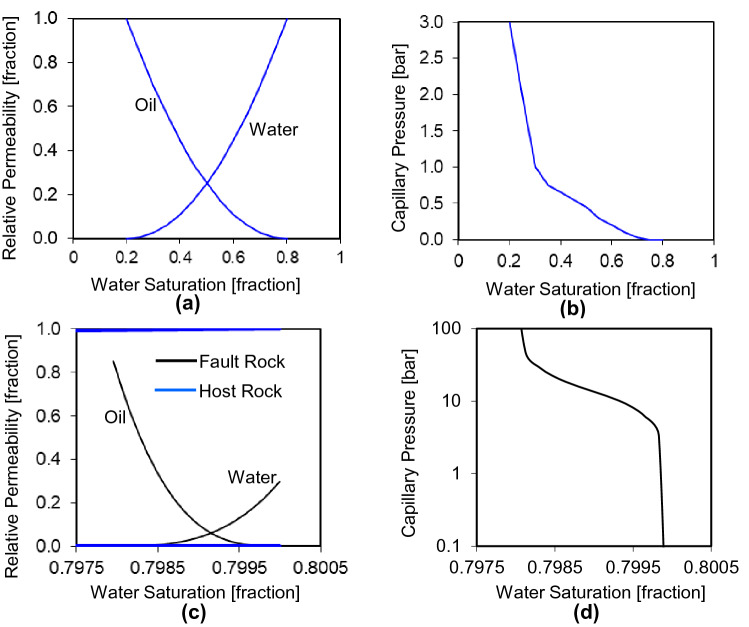
Input parameters of host rock and fault rock properties for the truth model. (**a**) The relative permeability and (**b**) the capillary pressure function of the host rock. (**c**) The relative permeability and (**d**) the capillary pressure curves of the fault rock.

The geometry of the truth and upscaled models were built using FaultMaker^21^ using the procedure explained by Islam and Manzocchi^10^. The sizes of the cells in the truth model are guided by the geometry of the fault zone components modeled and therefore are smaller near the fault (Fig. 2a). Cells in the upscaled model are all the same size. Cell permeabilities in horizontal directions (k_x_ = k_y_) range between about 10–1000 mD and k_v_/k_h_ ratio is 0.0001, porosity ranges about 3–50%, and net-to-gross ranges about 2–90%, based on a correlated random field modeled independently in each of the five layers using Schlumberger's Petrel software^22^. The oil and water relative permeability functions (Fig. 3a) for the unfaulted host rock cells follow those used in the 10th SPE Comparative Solution Project^23^. The capillary pressure curve is a user defined imbibition curve (Fig. 3b). Fault rock is assumed to be present on all fault surfaces with a permeability (*k*_f_) of 0.001 mD and a thickness (*t*_f_) modeled as a constant ratio of fault throw (i.e., *D*), using the expression, *t*_*f*_ = *D*/170. These single-phase fault rock properties are included in the truth model as transmissibility multipliers calculated in the TransGen software^24^ following the procedure defined in Fig. 1a. Oil and water relative permeability and imbibition capillary pressure functions for the fault rock (Fig. 3c,d) were defined using the type-curves defined by Manzocchi et al.^11^, for a connate water saturation of 0.8. As explained in introduction section, two-phase fault rock properties cannot be included directly in a flow simulation model without an explicit representation of the fault as grid-cells since these properties are tied to the water saturation of the fault rock. Hence, Manzocchi et al.^7^ devised a procedure for upscaling the two-phase fault rock properties and representing them as directional, irreversible pseudo-relative permeability curves attached to the cells upstream of faults (Fig. 1b). The implementation of the approach in the TransGen software^24^ and its application in full-field scale simulation models are described by Manzocchi et al.^11^. Application of the procedure in the present study is summarized in the remainder of this section.

**Figure 4:**
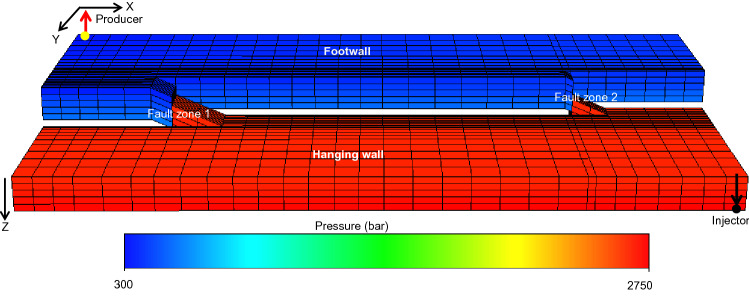
The pressure distribution in the truth model at water breakthrough time obtained from single-phase flow simulation using the well locations indicated.

The model is originally oil-saturated, and a water-flood using an injector-producer well pair is considered. The water injector is completed in the bottom layer of the hanging wall in one corner of the model, and the oil production well completed in the top layer of the opposite corner of the model **(**Fig. 4). The injector is controlled by a target fluid rate of 100 m^3^/d, and the producer well is controlled by fixed bottom-hole pressure of 300 bars. Average values of oil properties e.g., formation volume factor and viscosity are 1.03 rm^3^/sm^3^ and 2.875 cP respectively.

The pseudo-relative permeability curves used to include the fault rocks in the truth model are flow rate dependent, and the flow rate used in the procedure to calculate them (Fig. 2b) derive from a flow model using the correct geometry, well controls and single-phase fault rock properties as the truth model, but no two-phase properties^7,11^.The cell pressures observed in this model (Fig. 4) are used to define across-fault flow rates used in the two-phase fault rock property upscaling of the truth model.

**Figure 5:**
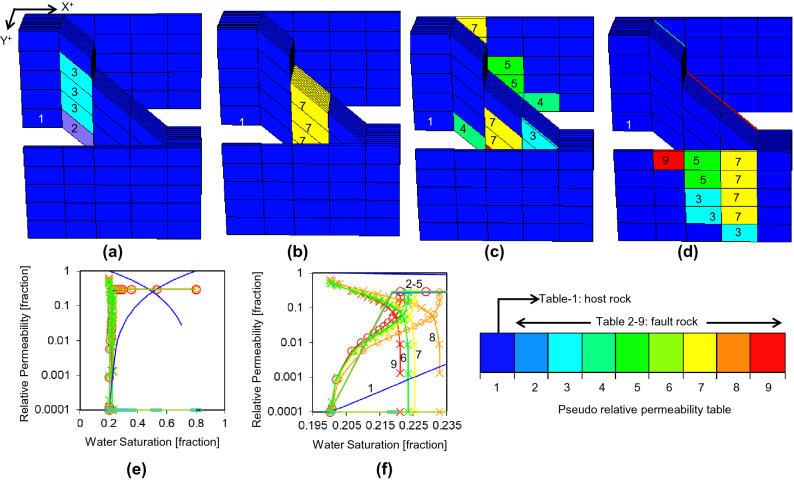
Indices for the directional, irreversible pseudo-relative permeability tables in the truth model for the X+ (**a**), X− (**b**), Y+ (**c**), and Y− (**d**) directions. (**e**) The host rock and eight pseudo-relative permeability functions shown with the same color scheme as (**a**–d). (**f**) As (**e**), but limited to the water saturation range in which the functions are variable. Crosses are for oil, circles are for water.

As well as flow rate, two other factors that influence the upscaled pseudo-relative permeability functions are variables in this study: the upstream grid-block permeability and the fault rock thickness. The pseudoization procedure in TransGen groups together faulted connections that share similar properties and creates an upscaled pseudo-relative permeability function for each group based on representative properties^11^. For the current study, eight groups have been created from the three variables and the resultant functions are shown for fault zone 1 in Fig. 5. The pseudo-functions are implemented in the truth model as directional and irreversible properties to the grid-cells adjacent to fault, and hence different pseudo-functions are used in each of the four directions (Fig. 5a–d). The upscaled pseudo-relative permeability functions have a small range of effective water saturation which is close to the irreducible water saturation of the parent grid block (Fig. 5e,f). This is because the method used to derive them^11^ assumes capillary pressure continuity across the interface between the host-rock and fault rock, and the high capillary pressure in the fine-grained fault rock are matched only at very low water saturations in the host-rock. These large change in flow property over a small saturation ranges means that small simulation time-steps are required, but if they are used carefully accurate flow simulation results can be obtained^7^.

## Summary of the modeling process

**Figure 6:**
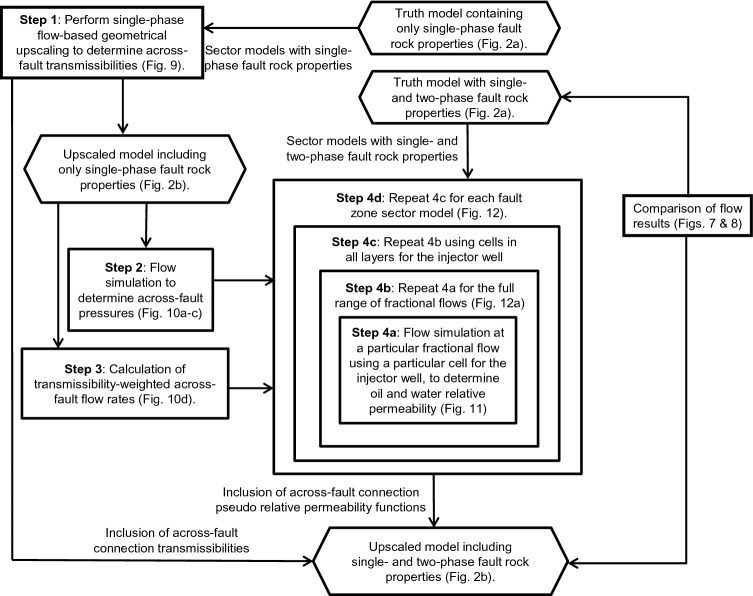
The workflow developed in this paper. Rectangles represent steps in the procedure, arrows record the transfer of information, and hexagons shows the four versions of the simulation model.

The principal innovation in this paper is the development of an upscaling method able to represent simultaneously the geometrical complexity and single- and two-phase petrophysical properties of the faults in the truth model (Figs. 2a and 5) within the low-resolution upscaled model in which the fault is represented as a planar discontinuity that completely offsets the reservoir sequence (Fig. 2b). The two-phase flow-based geometrical upscaling method that has been developed to do this is described step-by-step in the methods section at the end of the paper. This section provides an overview of the method and explains the construction of the four different models which are compared to each other in the following section. A work-flow diagram explaining the modeling is provided in Fig. 6.

The two versions of the truth model were introduced in the previous section. These models contain an explicit representation of the fault zone and therefore use the geometry shown in Fig. 2a. The single-phase truth model contains only fault transmissibility multipliers to represent fault rocks in this model. The two-phase truth model additionally contains the relative permeability and capillary pressure properties of the fault rocks, which (as explained in the previous Section) are included in the cells adjacent to the faults as directional irreversible pseudo-relative permeability functions (e.g., Fig. 5).

The overall objective of the current work is to create an upscaled model in which both single- and two-phase fault rock properties are represented within the simplified model geometry shown in Fig. 2b, and this upscaled model is the final product of the workflow summarised in Fig. 6 and explained in detail in the final section of the paper. Part of the workflow for creating this model involves the construction of an intermediate upscaled model using the same geometry as the final one (Fig. 2b), but which includes only single-phase fault rock properties. This model is created by Step 1 of the workflow (Fig. 6), using the single-phase flow-based geometrical upscaling method^10^.

**Figure 7:**
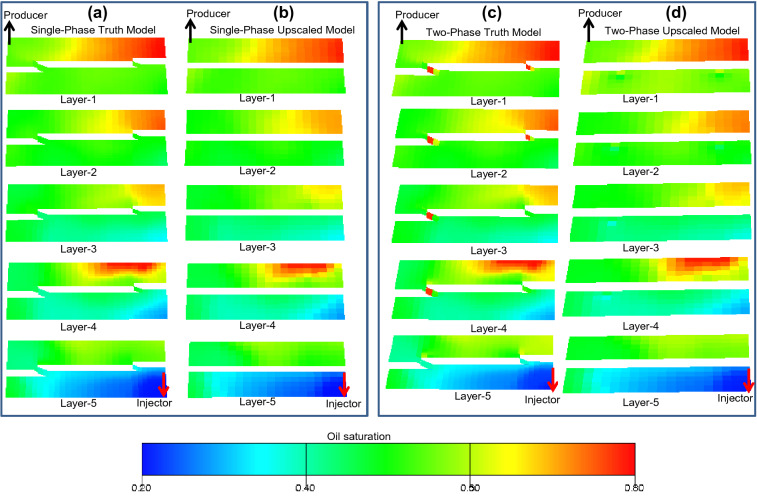
Oil saturation maps at 5 years for each layer of (**a**) the single-phase truth model, (**b**) the single-phase upscaled model, (**c**) the two-phase truth model, and (**d**) the two-phase upscaled model.

Hence, the modeling in the paper creates combinations of single-phase and two-phase, truth and upscaled models. A water-flood flow simulation using the wells shown in Fig. 4 with the well controls explained in the previous section has been run on each of these four model representations (e.g., Fig. 7). Quantitative results of these simulations are discussed in the following section, with the aim of establishing the accuracy of the new upscaling method. CPU run-times for the first 10 years of simulation were 117 s for the single-phase truth model (Fig. 7a), 96 s for the single-phase upscaled model (Fig. 7b), 145 s for the two-phase truth model (Fig. 7c), and 123 s for the two-phase upscaled model (Fig. 7d). These run-times indicate that the upscaling provides a modest improvement in performance, but improving performance is not the main motivation of this work as the workflow applied in this paper is not the same as the workflow that would be applied if the new upscaling method were to be used in a full-field flow simulation study. The objective of the current study is to describe the method and investigate its accuracy, and to do this requires comparisons between truth and upscaled models. In a full-field simulation study the simplest approach to apply the method developed would be to build and upscale sector models sequentially and independently, with no requirement to build high-resolution truth models of the full field or even each fault (e.g., Fig. 2a). This is impractical for realistic full-field models containing tens of faults and hundreds of thousands of grid-blocks. However, the sequential treatment of independent sector models to establish the upscaled properties as proposed here is similar to the procedure previously applied when implementing single-phase template-based geometrical upscaling in full-field flow simulation modeling^11^ but is beyond the scope of the current paper.

## Results and discussion

**Figure 8:**
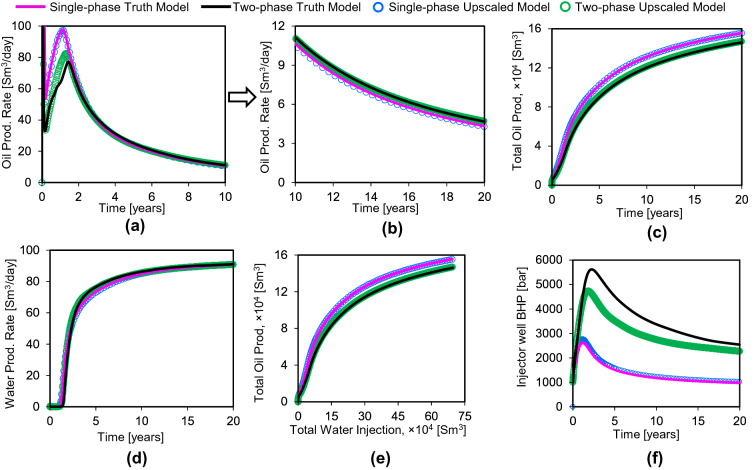
Flow responses of truth and upscaled models over the simulation time of 20 years. Oil production rate: (**a**) is for until 10 years of simulation and (**b**) is from 10 to 20 years of simulation for clarity. (**c**) Total oil production, (**d**) water production rate, (**e**) total oil production vs. total water injection to represent the sweep pattern, and (**f**) injector well bottom-hole pressure.

Flow simulation results for the four different models described above are shown in Fig. 8. The single-phase and two-phase truth models (Fig. 8) are illustrated by the pink and black curves respectively. It is important for the objectives of this study that there is a significant difference between the behavior of the truth model with and without two-phase fault rock properties, as this means that the two-phase aspects of the flow-based geometrical upscaling method can be tested. This is the reason why such a low fault permeability (i.e., 0.001 mD) has been used in this study: a higher absolute fault permeability would result is less difference in performance as a function of including two-phase fault rock properties and hence would not provide as rigorous a test of the two-phase upscaling.

A comparison of the behavior of the two truth models shows that the two-phase fault rock properties clearly influence production. Peak oil production rate occurs at about 1.5 years and is significantly lower in the two-phase case (Fig. 8a). At later times, oil production rates are slightly higher for the two-phase case (Fig. 8b). Water breakthrough occurs slightly later in the two-phase case but the subsequent increase in water production is more abrupt (Fig. 8d). For the same volume of injected water, oil production is consistently about 8% lower when two-phase fault rock properties are included compared to when they are not (Fig. 8e). Although the oil and water production rate curves show systematic differences in model behavior, the largest differences between the models are reflected in the bottom-hole pressure of the injector well (Fig. 8f). The injector well in these models is controlled by a constant flow rate, with its BHP allowed to vary to accommodate this rate and the constant BHP of the producer well (300 bars). This has resulted in unrealistically high injector well pressures for the models containing two-phase fault rock properties (Fig. 8f). In reality, the injector well would be unable to sustain its target fluid rate in these cases, leading to a reduction in injection rate at a lower BHP. However, since this model is an idealized illustrative example rather than a representation of a real reservoir, it has been convenient to allow the injector well BHP to rise to very large values so that differences in model behavior can be assessed as a function of one injector well variable (BHP) as opposed to two (BHP and rate). Results indicate that the pressure gradient between injector and producer required to sustain the target bottom-hole pressure is approximately 2.5 times greater when the two-phase fault rock properties are included compared to when they are not.

Step 2 of the two-phase upscaling relies on across-fault flow rates derived from a model upscaled using single-phase flow-based geometrical upscaling (Fig. 6). Flow results show an excellent match between this upscaled model (blue circles in Fig. 8) and the single-phase fault rock truth-model (pink curve). This match confirms that the single-phase flow-based upscaling approach is valid^10^.

The truth and upscaled models including both single- and two-phase properties are shown by the black curves and green circles respectively, in Fig. 8. There is a good agreement between the oil and water flow rates in these models (Fig. 8a–e) apart from the early oil production rate which is slightly too high in the upscaled model (Fig. 8a). The models are controlled by liquid injection rates, so it is inevitable that the total flow rates are the same, but it is nonetheless encouraging that the differences in phase-specific production observed in the single-phase and two-phase versions of the truth model are reproduced in the upscaled model. The bottom-hole pressure of the producer well is fixed and the largest inaccuracy in the upscaled model is the bottom-hole pressure of the injector well, which is the parameter with the greatest freedom in these experiments. Results (Fig. 8f) indicate that in the two-phase upscaled model, the same flow rates are achieved with a pressure difference between injector and producer that is consistently lower than the pressure difference required in the truth model. For example, at the end of the simulation period (i.e., 20 years), the pressure difference in the truth model is 2243 bars, while in the upscaled model it is 1992 bars. This inaccuracy is put into context by the comparison with the pressure difference in the single-phase model, which is about 700 bars for both of truth and upscaled models (Fig. 8f). Therefore, the two-phase upscaling has gone 84% of the way to honoring the truth model (since the difference between 1992 bars and 700 bars is 84% of the difference between 2243 bars and 700 bars).

This is the first implementation of two-phase flow-based geometrical upscaling and we consider an 84% accuracy of the method to be a very promising result, particularly given the complexity of the upscaling problem which involves capturing both the geometrical and petrophysical effects of the three-dimensional fault zones into an upscaled model. The results indicate that the upscaled faults are rather too permissive to flow (in particular, to flow of oil) when compared to the truth model. Interestingly, Manzocchi et al.^7^ observed a similar pattern of mismatch between their two-phase upscaled fault rock model and their truth model, which they attributed to the use of a single flow rate during the upscaling despite a lower total mobility early in the simulation run when the model is oil saturated. This led Manzocchi et al.^11^ to iteratively update the pseudo-functions over the course of the simulation run to try to account for this effect, but since the models used in that study were not compare to a truth case it is unclear whether the extremely complicated process applied resulted in greater accuracy or not. As well as flow rate, other factors that may have contributed to the error observed in the results (Fig. 8f) include the use of only seven fractional flow values during the upscaling, the decision to assign zero transmissibility to the least permissive across-fault flow paths, numerical dispersion, numerical resolution of the simulator, non-convergence of the solver, and the use of highly variable grid-block sizes during the upscaling simulations. We have not attempted systematically to investigate the source of the error to improve the accuracy of the upscaling, and the few modifications to the workflow that we have tested have not resulted in significant improvements. Hence, while recognizing that we have achieved only an 84% match to the truth model, we nonetheless consider that the study demonstrates the strong potential of the new upscaling method, and regard attempting to refine the method to improve its accuracy to be beyond the scope of the present study.

An important factor that should be considered in any attempts to apply this new method to a full-field scale model is its complexity. The workflow developed involves many different steps (Fig. 6), including the use of specialist software to generate high-resolution fault zone sector models and to derive the upscaled directional permeability functions used to represent the two-phase fault rock properties in these sector models (FaultMaker^21^ and TransGen^24^ have been used for these steps). Additionally, many hundreds of individual flow simulation models must be run to derive the two-phase upscaled properties of the sector models for inclusion in the final upscaled relative permeability functions that include both fault zone geometry and fault rock properties. None of these steps are particularly intensive computationally (e.g., the average run-time of the Step 4a simulation models are 5 s) but, because there are many steps and models, automating the workflow would be challenging.

## Conclusions

This study has defined and tested a procedure for upscaling the geometry of three-dimensional fault zones and the single-phase and two-phase petrophysical properties of associated fault rocks, to the resolution at which faults are generally represented in full-field flow simulation models of conventional clastic reservoirs (i.e., as zero-volume interfaces between grid-blocks). The new approach combines previously defined methods for (1) single-phase flow-based geometrical upscaling of fault zones and (2) two-phase upscaling of fault rocks. A series of flow simulation models are run at different fractional flow rates using injector and producer wells located in different model layers within sector models of the fault zone. Results of these models allow calculation of pseudo-relative permeability functions of across-fault non-neighbor connections as a function of the water saturation of the up-stream grid blocks. These upscaled two-phase functions supplement the transmissibility values calculated for the same flow paths using the single-phase flow-based geometrical upscaling approach. A comparison of the flow responses between a truth model and a model upscaled with the newly developed method shows that the proposed method provides an 84% solution to the problem, since a model including the two-phase upscaled properties is 84% closer to the truth model than it is to a model that includes only single-phase fault zone properties.

## Methods

The newly devised method for simultaneously upscaling the geometrical properties of fault zones and the two-phase petrophysical properties of fault rocks is explained step-by-step in this section. The method, shown schematically in Fig. 6, combines and develops upon the pseudoization method for two-phase fault rocks used to generate the high-resolution truth model explained above^11^_,_ and the single-phase flow-based geometrical upscaling method developed by Islam and Manzocchi^10^. Single-phase geometrical upscaling is concerned with determining the transmissibilities of flow paths through complex three-dimensional fault zones (Fig. 2a) and representing them in a low-resolution flow simulation model (Fig. 2b) as neighbor and non-neighbor connections. The present study is concerned also with determining their saturation-dependent phase-specific pseudo-relative permeabilities.

**Figure 9:**
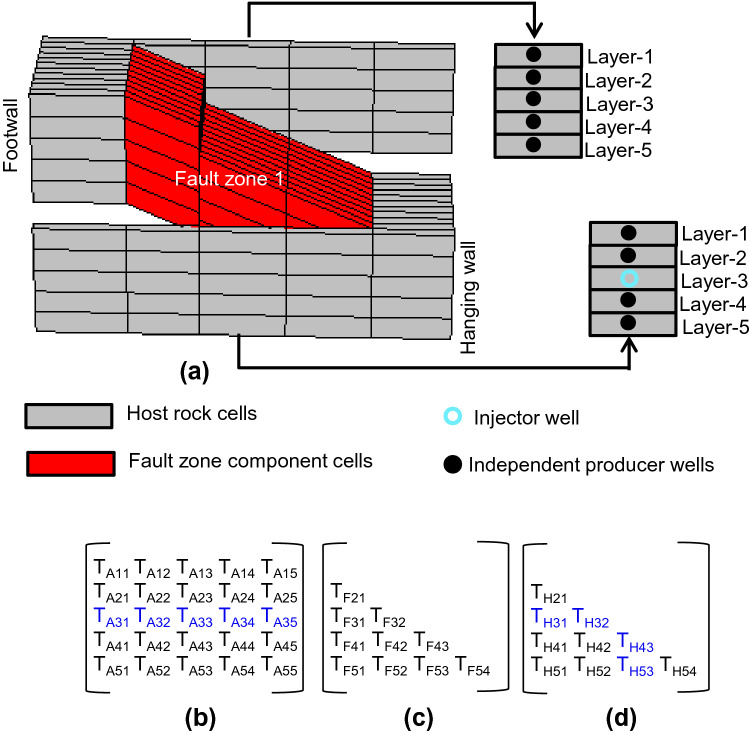
(**a**) Sector model of Fault zone 1, showing the well configurations used to calculate the transmissibilities of the flow paths indicated in blue for (**b**) across-fault flow, (**c**) flow between layers in the footwall, and (**d**) flow between layers in the hanging wall.

Step 1 (Fig. 6) is to perform a single-phase flow-based geometrical upscaling of the fault. This procedure, described in detail by Islam and Manzocchi^10^, results in the definition of a transmissibility value for each across-fault or along-fault flow paths present through each fault zone. For example, consider fault zone 1 (Fig. 9a). The dipping layers in the fault zone create multiple connections between different layers in the footwall and hanging wall, each of which may be an important flow path depending on the local boundary conditions and fluid distributions. The objective of geometrical upscaling is to calculate the transmissibility between different layers on both sides of the fault. The method focuses on individual layers, rather than individual cells, in the footwall and hanging wall. Hence although the sector model shown in Fig. 9 is five cells wide, upscaled transmissibilities are defined between the central cells in the footwall and hanging wall layers, as indicated by the labeled cells in Fig. 9a.

**Figure 10:**
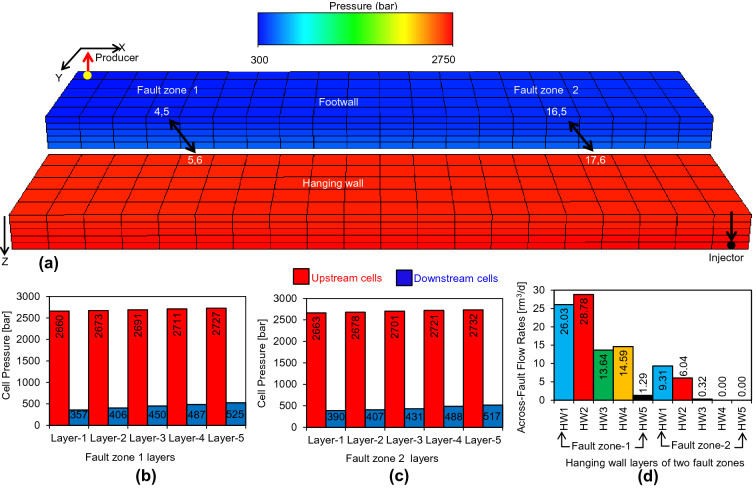
(**a**) Upscaled model with single-phase fault rock properties run through the flow simulator to measure across-fault cell pressures on either side of faults. (**b**) and (**c**) record the pressures of cells adjacent to fault zone 1 and 2 respectively and (**d**) shows the across-fault flow rates out from each hanging wall cell of both zones.

In this five-layer model, 45 possible transmissibilities exist through the fault zone (Fig. 9b–d): 25 across the fault (with transmissibilities labeled T_A_ in Fig. 9b) and 10 each between different layers in the footwall (T_F_ in Fig. 9c) and hanging wall (T_H_ in Fig. 9d). In single-phase flow-based geometrical upscaling^10^, these transmissibilities are calculated based on a series of steady-state, single-phase flow simulations conducted in a small-scale sector model local to the fault zone (Fig. 9a). Cells close to the center of the fault zone on both sides of the fault are chosen as separate well locations, and all vertical cell permeabilities are set to zero outside the fault zone (i.e., in the grey cells in Fig. 9a) to ensure that only flows through the fault zone are considered in the output transmissibilities. Each flow simulation is conducted using one well as water injector and the rest as water producers, and the transmissibilities of flow paths involving the injector cell are calculated from the simulation results using the procedures described in detail by Islam and Manzocchi^10^. For example, the 9 transmissibilities colored blue in Fig. 9b–d derived from the well configuration indicated in Fig. 9a. Changing the layer used for the injector well provide a different set of transmissibilities and for this 5-layer model, 10 simulations are required to derive all possible transmissibilities for this fault zone. Another 10 are needed for fault zone 2, so overall 20 flow simulations provide the results of the single-phase flow-based geometrical upscaling.

In Step 2 (Fig. 6), a flow simulation is made using the upscaled model (Fig. 2b) including the across-fault connection transmissibilities calculated in Step 1 to represent the single-phase flow properties of the fault zones. The purpose of this model (Fig. 10a) is to deduce pressure boundary conditions for the different flow paths across the fault. These are required for subsequent steps in the process, since the two-phase flow-based geometrical upscaling is flow rate dependent. The time of first water break through into the producer well is considered representative, and the pressures in the cells at the ends of the upscaled flow paths are extracted from the simulation results at this time (Fig. 10b,c).

The total across-fault flow rates (i.e., q = 100 m^3^/d, as defined by the overall model) and the individual across-fault transmissibilities calculated for each flow path in Step 1 (Fig. 9b) are used to determine transmissibility-weighted across-fault flow rates out of the 10 cells on the upstream side of the two fault zones (Fig. 10d). This calculation defines Step 3 of the overall process (Fig. 6), explained with reference to Layer 3 in the hanging wall of fault zone 1 (i.e., the reference layer in Fig. 9). If *q*_3_ is the transmissibility-weighted across-fault flow rate out of this cell and *T*_3_ is the cumulative across-fault transmissibility of all across-fault flow paths out of this cell (i.e., T_3_ is equal to sum of T_A31_, T_A32_, T_A33_, _TA34_, and T_A35_ in Fig. 9b), then q_3_ is given by:

1 $${q}_{3}=\frac{q.{T}_{3}}{\sum_{\mathrm{n}=1}^{10}{T}_{\mathrm{n}}}$$

In this equation, T_n_ refers to the 10 footwall cells associated with across-fault flow (five in each of fault zones 1 and 2). Results of Step 3 are shown in Fig. 10d.

The transmissibility-weighted across-fault flow rates and cell pressures calculated in Steps 2 and 3 are used in the two-phase flow-based geometrical upscaling to calculate the upscaled pseudo-functions in Step 4 (Fig. 6). This process is an extension of the single-phase upscaling procedure summarized in Step 1 (and discussed comprehensively by Islam and Manzocchi^10^). The main difference is that instead of running one single-phase simulation model to deduce the transmissibilities of multiple flow paths out of a single cell (as outlined in Fig. 9), several two-phase flow simulation models (each with a different fractional flow of oil and water) are required to determine the upscaled across-fault pseudo-relative permeability functions for the flow paths. The nested and recursive nature of the new method is represented by the concentric rectangles in the work-flow summary diagram (Fig. 6) which separate the four parts of the process (Steps 4a-d).

**Figure 11:**
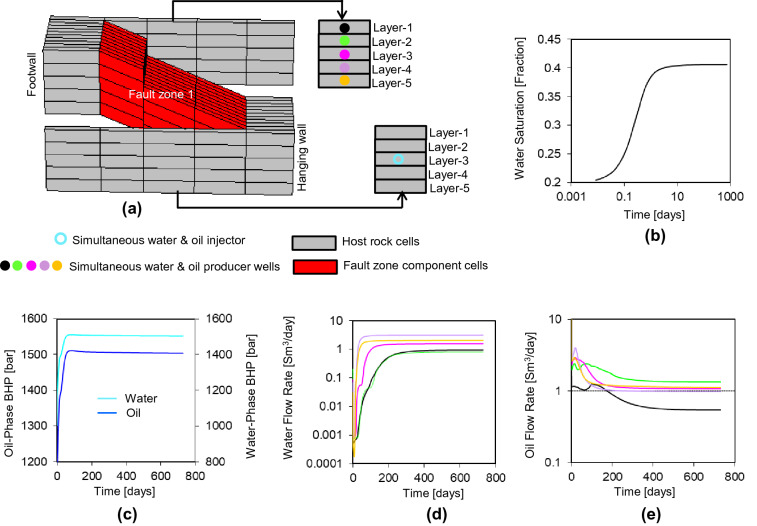
(**a**) As Fig. 9a, but showing the wells in the same colors as used in the remainder of the figure which shows simulation results for injection with a 40% fractional water flow. (**b**) Water saturation. (**c**) Oil- and water-phase injection well bottom-hole pressure. (**d**) Water and (**e**) oil flow rates. Note that the producer wells and associated data are not shown in the cells in layers 1, 2, 4 and 5 of the hanging wall, as flow rates into these wells were below the resolution of the simulator.

Step 4a consists of a flow simulation using a fault zone sector model which considers a single upstream cell and a single fractional flow. For example, Fig. 11 illustrates the case of a fractional flow of 40% water and 60% oil from the central layer of the hanging wall of fault zone 1. An injector well is placed in the upstream cell and independent producing wells are placed in the other layers on both sides of the fault (Fig. 11a). Simultaneous injection of oil and water at the specific fraction flow of interest is modeled with a combined injection rate equal to the across-fault flow rates for the cell calculated in Step 3 (Fig. 10d). Each of the producing wells is controlled by a target cell pressure equal to the one derived in Step 2 (Fig. 10b). The initial pressure of the injector well is also set to the value observed in Step 2, but is allowed to change over the course of the simulation (Fig. 11c) to honor the main control on the injector well which is its flow rates. The reason for an over-riding flow rate, rather than pressure, control on the injector well is because it is impossible in the simulator to define a specific fractional flow if a well is defined by pressure rather than flow rate.

Once flow has stabilized (after about 400 days for the case illustrated in Fig. 11), the water and oil flow rates observed in each across-fault flow path (*q*_w_ and *q*_o_ respectively, Fig. 11d,e) are used in conjunction with the phase-specific pressure difference between the injector and producer cells ($\Delta {p}_{\mathrm{w}}$ and $\Delta {p}_{\mathrm{o}}$), the water and oil viscosity (*µ*_*w*_ and *µ*_*o*_ respectively), and the across-fault transmissibility (*T*_*3*_) calculated in Step 1, to determine water and oil pseudo-relative permeability values (${k}_{\mathrm{rw}}^{{{\prime}}}$ and ${k}_{\mathrm{ro}}^{{{\prime}}}$) for the connection based on the multi-point flux approximation^25–28^ as follows:

2 $${k}_{\mathrm{rw}}^{{{\prime}}}=\frac{{q}_{\mathrm{w}}{\mu }_{\mathrm{w}}}{{T}_{3}\Delta {p}_{\mathrm{w}}}$$

3 $${k}_{\mathrm{ro}}^{{{\prime}}}=\frac{{q}_{\mathrm{o}}{\mu }_{\mathrm{o}}}{{T}_{3}\Delta {p}_{\mathrm{o}}}$$

**Figure 12:**
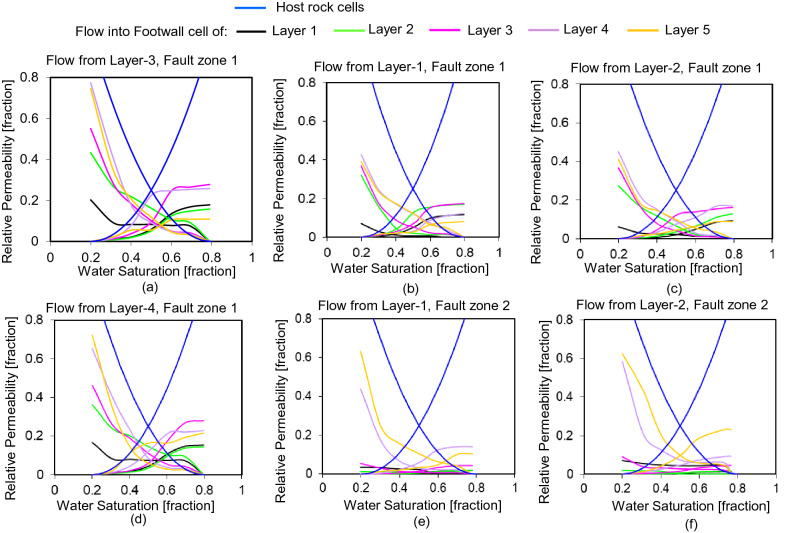
The upscaled pseudo-relative permeability curves. Each graph is associated with flow from the different hanging wall layers indicated, and the different colored curves are for flow into different footwall layers.

These pseudo-relative permeability values relate to the specific water saturation value of 0.4 defined in the upstream cell (Fig. 11b). Repetition of the process at different fractional flow values (Step 4b, Fig. 6) provide different values of ${k}_{\mathrm{rw}}^{{{\prime}}}$ and ${k}_{\mathrm{ro}}^{{{\prime}}}$ related to a different water saturation values in the upstream cell. For example, Fig. 12a shows the complete pseudo-relative permeability functions for the five across-fault connections associated with flow from the footwall cell of Layer 3 of fault zone 1 (i.e., the case shown in Fig. 11a), defined from seven flow simulations at water saturation values raging between 0.2 and 0.8 (the connate water saturation and water saturation at irreducible oil respectively) at saturation intervals of 0.1. Manipulations are required close the end-point saturations to achieve monotonic curves with zero values of ${k}_{\mathrm{rw}}^{{{\prime}}}$ and ${k}_{\mathrm{ro}}^{{{\prime}}}$ at the connate water and irreducible oil saturations respectively. Functions are only derived for the flow paths into the footwall layers, because sustainable flow rates from this hanging wall cell into the other hanging wall layers could not be maintained by the simulator and the producer wells in these cells became inactive. These connections are discussed further below.

The process described above provides a separate pseudo-relative permeability function for each across-fault flow path from the cell on the upstream side of the fault that contains the injector well (i.e., layer 3 of fault zone 1 for the case illustrated in Figs. 11 and 12a). In Step 4c (Fig. 6), further sets of flow simulation models are run using different layers for the injector cell to provide the set of pseudo-relative permeability curves for across-fault flow paths associated with that fault zone (Fig. 12b–d). In Step 4d (Fig. 6), the process is repeated for the second fault zone. Hence, a systematic treatment of all layers for both fault zones provides the full set of pseudo-relative permeability functions associated with each across-fault flow path present in the truth model. These functions can be specified individually for each neighbor and non-neighbor connection in the upscaled model in conjunction with the connection transmissibility. Therefore, this two-phase flow-based geometrical upscaling process results in a reproduction in the upscaled model (Fig. 2b) of geometrical and both single-phase and two-phase fault rock effects present in the truth model (Fig. 2a). The accuracy of the upscaling is discussed by comparing the performance of the truth and upscaled models in the results section. A few aspects of two-phase flow-based upscaling and its output which might contribute to the imperfect match between these models discussed in the remainder of this section.

In principle, the flow-based geometrical upscaling of the example model should provide a unique transmissibility value (from the single-phase upscaling) and pseudo-relative permeabilities functions (from the two-phase upscaling) for 50 across-fault flow paths (25 for each of the two fault zones, Fig. 9b) as well as 40 flow paths between cells on one or other sides of the fault (half of these flow paths, associated with one fault zone, are shown in Fig. 9c,d). In practice, however, the transmissibilities of some of these connections are so low that the flow rates fall below the numerical resolution of the simulator. This can occur either during the single-phase upscaling, in which case the potential connections are considered to be absent and are not considered further, or during the two-phase upscaling, in which case both the oil and water relative permeabilities are set to zero for the entire saturation range. Hence, there are no transmissibilities associated with any of the along-fault connections (T_F_ and T_H_, Fig. 9) as the pressure difference between cells on a particular side of the fault is too low (Fig. 10b,c). Additionally, no transmissibilities are calculated for Layers 4 and 5 of fault zone 2, since flow rate out of these cells is very low (Fig. 10d) due to locally low permeabilities (Fig. 2). Of the remaining across-fault connections, those associated with hanging wall Layer 5 of fault zone 1 and hanging wall Layer 3 of fault zone 2, are assigned zero values of ${k}_{\mathrm{rw}}^{{{\prime}}}$ and ${k}_{\mathrm{ro}}^{{{\prime}}}$ as they cannot sustain adequate flow rates in the two-phase simulations. The result of the two-phase upscaling, therefore, are pseudo-relative permeability functions for the 30 most transmissible across-fault flow paths from six of the upstream hanging wall cells of the two fault zones (Fig. 12).

As well as calculating upscaled pseudo-relative permeability functions for each connection out of the upstream cell containing the injector well, the two-phase flow-based geometrical upscaling method is capable of calculating an upscaled pseudo-capillary pressure function for the cell following the Kyte and Berry^29^ approach, as the oil-phase (e.g., non-wetting) and water-phase (e.g., wetting) pressures in this cell are known (Fig. 11c). However, unlike relative permeability for which a different function can meaningfully be assigned for each flow path out of a particular cell, capillary pressure is not a directional property and only one capillary pressure curve can be defined per cell. Previous considerations of this issue^7,30^ have concluded that it is most appropriate to use the original unaltered capillary pressure curve rather than an upscaled one, and this is the approach taken in this study.

## Data Availability

The datasets that support plots and other findings of this study are available from the corresponding author upon reasonable request.
